# Beijing Lineage of MDR *Mycobacterium tuberculosis* in Bulgaria, 2007–2011

**DOI:** 10.3201/eid2011.140468

**Published:** 2014-11

**Authors:** Stefan Panaiotov, Elizabeta Bachiyska, Stanislava Yordanova, Yuliana Atanasova, Nadia Brankova, Viktoria Levterova, Sarah Sengstake, Richard Anthony, Indra Bergval, Christophe Sola, Todor Kantardjiev

**Affiliations:** National Center of Infectious and Parasitic Diseases, Sofia, Bulgaria (S. Panaiotov, E. Bachiyska, S. Yordanova, Y. Atanasova, N. Brankova, V. Levterova, T. Kantardjiev);; Royal Tropical Institute, Amsterdam, the Netherlands (S. Sengstake, R. Anthony, I. Bergval);; University Paris-Sud, Orsay, France (C. Sola)

**Keywords:** tuberculosis, MDR TB, XDR TB, genotype, tuberculosis and other mycobacteria, Bulgaria, Beijing lineage

## Abstract

To assess the spread of the *Mycobacterium tuberculosis* Beijing genotype among patients with multidrug-resistant and extensively resistant tuberculosis in Bulgaria, we genotyped 188 (72%) of 261 microbiologically confirmed resistant isolates obtained during 2007–2011. The estimated prevalence of the Beijing genotype among these patients was 3.2%.

In 1995, the Beijing genotypic family was identified in Beijing, China, where it accounted for at least 86% of *Mycobacterium tuberculosis* isolates (*1*). Genotyping of 2,092 multidrug resistant (MDR) and extensively drug-resistant (XDR) strains, isolated in 24 European countries during 2003–2011, indicated that 470 (22.5%) strains belonged to the Beijing lineage, many of which originated from eastern European countries (*2*). Strains of this genotypic family account for at least 13% of *M. tuberculosis* strains worldwide, and the percentage seems to be increasing (*3*,*4*).

Bulgaria is 1 of 27 countries that the World Health Organization describes as having a high burden of MDR tuberculosis (TB). TB case notification rates in Bulgaria were 39.6 cases/100,000 population (3,038 cases) in 2007, 41.2 cases/100,000 population (3,150 cases) in 2008, 38.3 cases/100,000 population (2,910 cases) in 2009, 35 cases/100,000 population (2,649 cases) in 2010, and 32.1 cases/100,000 population (2,407 cases) in 2011. During this same period, the MDR/XDR TB notification rate was 7.5% (76 cases) in 2007, 3.3% (31 cases) in 2008, 5.1% (43 cases) in 2009, 5.8% (56 cases) in 2010, and 7.5% (55 cases) in 2011 (*5*).

In a nationwide study of 197 drug-susceptible isolates obtained during 2003–2004, no isolates of the Beijing genotype were identified in Bulgaria (*6*). A subsequent study also did not identify the Beijing genotype among 133 drug-resistant and drug-susceptible *M. tuberculosis* isolates obtained during 2004–2006 (*7*). Since then, Beijing strains of *M. tuberculosis* have been identified during routine screening of MDR/XDR TB isolates in Bulgaria. In this study, after receiving ethics approval, we assessed the spread of the *M. tuberculosis* Beijing genotype among patients with MDR and XDR TB in Bulgaria.

## The Study

In Bulgaria during 2007–2011, a total of 188 MDR/XDR *M. tuberculosis* isolates were characterized by drug-susceptibility testing and spoligotyping (31 in 2007, 31 in 2008, 39 in 2009, 47 in 2010, and 40 in 2011) and represent 72% of the 261 MDR/XDR *M. tuberculosis* isolates identified during that period. The MDR/XDR strains were isolated from sputum of 181 (96.2%) patients, gastric lavage fluid from 3 (1.5%), bronchoalveolar lavage fluid from 2 (1%), pleural fluid from 1 (0.5%), and fistula swab sample from 1 (0.5%). The first MDR/XDR *M. tuberculosis* strain isolated per patient was analyzed for this study. 

Drug susceptibility of these strains was confirmed by the National Reference TB Laboratory, which used liquid culture at concentrations of 0.1 μg/mL for isoniazid, 1 μg/mL for rifampin, 5 μg/mL for ethambutol, 1 μg/mL for streptomycin, 2.5 μg/mL for capreomycin, 1 μg/mL for amikacin, 5 μg/mL for kanamycin, and 2 μg/mL for ofloxacin. Of the MDR/XDR strains, 77 (41%) were resistant to all first-line anti-TB drugs, 51 (27%) were resistant to isoniazid and rifampin; 38 (20%) to isoniazid, rifampin, and streptomycin; and 22 (12%) to isoniazid, rifampin, and ethambutol. Second-line drug-susceptibility testing was performed for 174 (81%) of the MDR strains. Of these, 140 (80%) were sensitive to all second-line anti-TB drugs and 20 (12%) were resistant to ofloxacin. Five percent (n = 9) of XDR strains had combined resistance to ofloxacin, amikacin, kanamycin, and capreomycin.

To detect and genotype the Beijing strains, we used a spoligotyping kit (Isogen Bioscience BV, Maarssen, the Netherlands), and we performed single-nucleotide polymorphism typing by bead-based multiplex ligation-dependent probe amplification (MLPA) (*8*,*9*). We also screened for the presence of the Beijing genotype in 117 drug-sensitive strains collected from across the country in 2011, representing a convenience sample of ≈10%. Both methods identified 1 drug-sensitive *M. tuberculosis* strain of the Beijing genotype (BG_112_11, Table 1). The MLPA test assigned this isolate to the Beijing K1 sublineage (*10*).

**Table 1:** Phenotypic characteristics of the *Mycobacterium tuberculosis* Beijing genotype strains identified in Bulgaria, 2007–2011*

Strain	Patient origin	Patient age ,y/sex	Case status	Drug susceptibility	First-line drugs					Second-line drugs			
					STR	INH	RIF	EMB		OFL	AMK	KAN	CAP
BG_07_09	Dobrich	35/M	PT	MDR	R	R	R	R		S	S	S	S
BG_35_09	Sofia	42/M	New	MDR	R	R	R	R		S	S	S	S
BG_104_10	Sofia	25/F	New	XDR	R	R	R	R		R	R	R	R
BG_85_10	Varna	32/M	New	MDR	R	R	R	R		S	S	S	S
BG_95_10	Varna	59/M	New	MDR	R	R	R	R		S	S	S	S
BG_112_11	Sofia	32/M	New	Susceptible	S	S	S	S		NA	NA	NA	NA
BG_54_11	Sofia	48/M	PT	MDR	R	R	R	R		S	S	S	S

*AMK, amikacin; CAP, capreomycin; EMB, ethambutol; INH, isoniazid; KAN, kanamycin; NA, not applicable; OFL, ofloxacin; PT, previously treated; R, drug resistant; RIF, rifampin; S, drug sensitive; STR, streptomycin..

Among the 188 MDR/XDR strains collected during 2007–2011, a total of 6 isolates with the Beijing genotype were identified (prevalence 3.2%, 95% CI 0.7%–5.7%): 2 in 2009 (Beijing SA+/CHIN+/V+), 3 in 2010 (Beijing SA+/CHIN+/V+ and Beijing K1), and 1 in 2011 (Beijing SA+/CHIN+/V+). These 6 drug-resistant strains were isolated from 5 male and 1 female patients. Drug-susceptibility testing and molecular drug-resistance markers confirmed that 1 strain was XDR. A total of 4 strains were Spoligo International Type 1 (SIT1) and 2 strains were SIT265 (Tables 1 and 2). In the neighboring countries of Albania, Greece, Romania, and Turkey, no MDR *M. tuberculosis* Beijing strains were isolated or prevalence was 1%–3%, mostly of imported origin (*11*–*14*). The origins of the MDR/XDR strains and the drug-sensitive strain identified in Bulgaria are described below. Additional methods used and genotyping characteristics found are described in the Technical Appendix.

**Table 2:** Genotyping characteristics of the *Mycobacterium tuberculosis* Beijing genotype strains identified in Bulgaria, 2007–2011*

Strain	Spoligotype pattern	SIT	24 MIRU-VNTR†	MLPA lineage type‡
BG_07_09	⬜⬜⬜⬜⬜⬜⬜⬜⬜⬜⬜⬜⬜⬜⬜⬜⬜⬜⬜⬜⬜⬜⬜⬜⬜⬜⬜⬜⬜⬜⬜⬜⬜⬜⬛⬛⬛⬛⬛⬛⬛⬛⬛	1	244233352634425153353823	SA+/V+/ CHIN+
BG_35_09	⬜⬜⬜⬜⬜⬜⬜⬜⬜⬜⬜⬜⬜⬜⬜⬜⬜⬜⬜⬜⬜⬜⬜⬜⬜⬜⬜⬜⬜⬜⬜⬜⬜⬜⬛⬛⬛⬛⬛⬛⬛⬛⬛	1	244233352644425173343723	SA+/V+/ CHIN+
BG_104_10	⬜⬜⬜⬜⬜⬜⬜⬜⬜⬜⬜⬜⬜⬜⬜⬜⬜⬜⬜⬜⬜⬜⬜⬜⬜⬜⬜⬜⬜⬜⬜⬜⬜⬜⬛⬛⬛⬛⬛⬛⬛⬛⬛	1	244233352644425153353823	SA+/V+/ CHIN+
BG_85_10	⬜⬜⬜⬜⬜⬜⬜⬜⬜⬜⬜⬜⬜⬜⬜⬜⬜⬜⬜⬜⬜⬜⬜⬜⬜⬜⬜⬜⬜⬜⬜⬜⬜⬜⬛⬛⬜⬛⬛⬛⬛⬛⬛	265	244233352644425153353823	K1
BG_95_10	⬜⬜⬜⬜⬜⬜⬜⬜⬜⬜⬜⬜⬜⬜⬜⬜⬜⬜⬜⬜⬜⬜⬜⬜⬜⬜⬜⬜⬜⬜⬜⬜⬜⬜⬛⬛⬜⬛⬛⬛⬛⬛⬛	265	244233352644425153353823	K1
BG_112_11	⬜⬜⬜⬜⬜⬜⬜⬜⬜⬜⬜⬜⬜⬜⬜⬜⬜⬜⬜⬜⬜⬜⬜⬜⬜⬜⬜⬜⬜⬜⬜⬜⬜⬜⬛⬛⬛⬛⬛⬛⬛⬛⬛	1	NA	K1
BG_54_11	⬜⬜⬜⬜⬜⬜⬜⬜⬜⬜⬜⬜⬜⬜⬜⬜⬜⬜⬜⬜⬜⬜⬜⬜⬜⬜⬜⬜⬜⬜⬜⬜⬜⬜⬛⬛⬛⬛⬛⬛⬛⬛⬛	1	244233352634425153353823	SA+/V+/ CHIN+

*MIRU, mycobacterial interspersed repetitive unit; NA, not applicable; SIT, Spoligo International Type second to SITVITWEB database classification; VNTR, variable number–tandem repeat. †24 loci MIRU-VNTR order: 154, 424, 577, 580, 802, 960, 1644, 1955, 2059, 2163b, 2165, 2347, 2401, 2461, 2531, 2687, 2996, 3007, 3171, 3192, 3690, 4052, 4156, 4348. ‡SA+, South Africa; V+, Vietnam; CHIN+, China; K1, Uzbekistan (*9*,*10*).

The first XDR Beijing strain identified (BG_104_10) was from a patient who arrived in Bulgaria from Moldova in 2009 and in whom TB developed a few months later. Before coming to Bulgaria, the patient had had contact with a TB patient in Ukraine. The first *M. tuberculosis* isolate recovered from this Moldovan patient was confirmed as primary XDR *M. tuberculosis*. The patient received treatment with second-line TB drugs in 2010, underwent surgery, and in 2011 was considered cured.

The 2 SIT265 Beijing genotype clinical isolates (BG–85-10 and BG–95–10) originated from 2 neighbors. One patient was an alcohol-dependent sailor in whom TB developed after a ship voyage. TB developed in his neighbor (who had diabetes) a few months later. *M. tuberculosis* transmission most likely occurred between them. The epidemiologic link was supported by identical drug-susceptibility testing results, MLPA, spoligotyping, and variable number–tandem repeat typing profiles with no similarities to other genotypes identified in Bulgaria. Most of the 50 SIT265 Beijing genotype strains, reported in the SITVITWEB database (http://www.pasteur-guadeloupe.fr:8081/SITVIT_ONLINE), originated from the United States, Russia, Israel, and Spain (*4*). Both patients were receiving treatment as of 2012.

The other 3 MDR Beijing isolates had different VNTR patterns but shared the same SIT1 spoligotype and the same MLPA lineage type (SA+/V+/CHIN+) (Table 2). Of these 3 patients, 2 lived in the capital city of Sofia (with strains BG_54_11 and BG_112_11) and the third (with strain BG_07_09) lived in Dobrich, in the northeastern part of the country. The patient was alcoholic, antisocial, and homeless and refused treatment. The MDR TB was diagnosed in 2007, but the strain was lost. In 2009, the patient was hospitalized, and Beijing genotype MDR *M. tuberculosis* was identified. The patient died that same year.

Another Beijing strain was identified in 2009 from a patient who lived in Sofia (BG_35_09, Tables 1 and 2). The patient’s parents were from Armenia, where the prevalence of the Beijing genotype is no less than 41% (*4*) and prevalence of MDR TB in 2009 was 22.9% (*5*). The patient had not been to Armenia, but the possibility of casual contact with migrating Armenians was not excluded. This patient died in 2010.

## Conclusions

Among MDR/XDR *M. tuberculosis* isolates in Bulgaria, prevalence of the Beijing genotype is low. Not all detected cases of TB caused by the Beijing genotype were a result of human migration; MDR/XDR TB transmission within the country was also observed. The *M. tuberculosis* Beijing genotype strains are considered to be large drivers of international TB transmission and are associated with the emergence and spread of MDR/XDR TB (*2*). This finding demands organization of wider surveillance in Bulgaria that includes monitoring genotypes of drug-susceptible and drug-resistant *M. tuberculosis* strains.

Technical Appendix Additional methods used and genotyping characteristics found for the identified *Mycobacterium tuberculosis* Beijing strains isolated in Bulgaria during 2007–2011.
