# Supplementary material for: Beijing Lineage of MDR Mycobacterium tuberculosis in Bulgaria, 2007–2011
Source: Emerg Infect Dis. 2014 Nov;20(11):1899–901. doi: 10.3201/eid2011.140468 (PMC4214304; doi:10.3201/eid2011.140468)
Supplement: Technical Appendix — Additional methods used and genotyping characteristics found for the identified Mycobacterium tuberculosis Beijing strains isolated in Bulgaria during 2007–2011. [file 14-0468-Techapp-s1.pdf]

Article DOI: http://dx.doi.org/10.3201/eid2011.140468

Beijing Lineage of MDR *Mycobacterium tuberculosis* in Bulgaria, 2007–2011

Technical Appendix

A strain was defined as Beijing if the RD105 region was deleted and if an additional Beijing marker was present: RD131, pckA, fbpB-238 and mutT2–58 (*1*) (Technical Appendix Table). The China (CHIN+ and CHIN–), Vietnam (V+ and V–), Uzbekistan (K1 and K2) and South Africa (SA+ and SA–) genomic sublineages of Beijing strains (*2*) were discriminated according to the following scheme: Atypical Beijing strains belonging to the South Africa (SA–) or China (CHIN–) sublineage were identified by the presence of markers RD105 and pckA. Atypical Beijing strains belonging to the Vietnam (V–) sublineage were identified by the presence of RD105 and fbpB-238. Typical Beijing strains belonging to the South Africa (SA+) China (CHIN+), Vietnam (V+) or the Uzbekistan (K2) sublineage were identified by the presence of the markers RD105, fbpB-238 and mutT2–58. The Beijing strains belonging to the Uzbekistan (K1) sublineage were identified by the presence of markers RD105, fbpB-238, mutT2–58 and RD131 (*1*,*2*). MLPA analysis confirmed the identified Beijing spoligotype. In addition, the Beijing genotype of one pansusceptible Beijing strain (BG_112_11) was also confirmed by MLPA. Strains BG_07_09, BG_35_09, BG_104_10 and BG_54_11 were defined as SA+ or V+ or CHIN+, while BG_85_10, BG_95_10 and BG_112_10 belong to K1 sublineage which accounts for a significant proportion of the Beijing genotype strains in Europe and is associated with a large European MDR cluster (*1*,*3*).

Technical Appendix Table. Genotyping characteristics of the identified *Mycobacterium tuberculosis* Beijing strains isolated in Bulgaria, 2007–2011*

		Markers specific for the Beijing clade					
Strain	MLPA lineage type†	mutT2–58	fbpB-238	acs-1551	RD131 (E0054 cluster (*4*)	RD105	pckA-1119
BG_07_09	SA+/V+/ CHIN+	+	+	+	–	+	–
BG_35_09	SA+/V+/ CHIN+	+	+	+	–	+	–
BG_104_10	SA+/V+/ CHIN+	+	+	+	–	+	–
BG_85_10	K1	+	+	+/–‡	+	+	–
BG_95_10	K1	+	+	+/–‡	+	+	–
BG_112_11	K1	+	+	+/–‡	+	+	–
BG_54_11	SA+/V+/ CHIN+	+	+	+	–	+	–

*Isolation by bead-based multiplex ligation-dependent probe amplification.
†SA+, South Africa; V+, Vietnam; CHIN+, China; K1, Uzbekistan (*1*,*2*).
‡The threshold value is not very well defined for this marker.
.

Technical Appendix References

1. Bergval I, Sengstake S, Brankova N, Levterova V, Abadía E, Akhalaia M, et al. Combined speciation, genotyping, and drug resistance detection of M. tuberculosis cultures by MLPA on a bead-based liquid array. PLoS ONE. 2012;7:e43240. PubMed http://dx.doi.org/10.1371/journal.pone.0043240.

2. Schürch AC, Kremer K, Hendriks ACA, Freyee B, McEvoy CRE, van Crevel R, et al. SNP/RD typing of Mycobacterium tuberculosis Beijing strains reveals local and worldwide disseminated clonal complexes. PLoS ONE. 2011;6:e28365. PubMed http://dx.doi.org/10.1371/journal.pone.0028365

3. De Beer JL, Ködmön C, van der Werf MJ, van Ingen J, van Soolingen D. the ECDC MDR-TB molecular surveillance project participants. Molecular surveillance of multi- and extensively drug-resistant tuberculosis transmission in the European Union from 2003 to 2011. Euro Surveill. 2014;19:20742. PubMed

4. Devaux I, Kremer K, Heersma H, Van Soolingen D. Clusters of multidrug-resistant *Mycobacterium tuberculosis* cases, Europe. Emerg Infect Dis. 2009;15:1052–60 [cited 2013 Mar 19]. http://wwwnc.cdc.gov/eid/article/15/7/08-0994
